# New Insights into Curcumin- and Resveratrol-Mediated Anti-Cancer Effects

**DOI:** 10.3390/ph14111068

**Published:** 2021-10-22

**Authors:** Andrea Arena, Maria Anele Romeo, Rossella Benedetti, Laura Masuelli, Roberto Bei, Maria Saveria Gilardini Montani, Mara Cirone

**Affiliations:** 1 Department of Experimental Medicine, “Sapienza” University of Rome, Laboratory Affiliated to Istituto Pasteur Italia-Fondazione Cenci Bolognetti, Viale Regina Elena 324, 00161 Rome, Italy; a.arena@uniroma1.it (A.A.); mariaanele.romeo@uniroma1.it (M.A.R.); rossella.benedetti@uniroma1.it (R.B.); laura.masuelli@uniroma1.it (L.M.); mariasaveria.gilardinimontani@uniroma1.it (M.S.G.M.); 2 Department of Clinical Science and Translational Medicine, “Tor Vergata” University of Rome, Via Montpellier 1, 00133 Rome, Italy; bei@med.uniroma2.it

**Keywords:** Her-2/neu cancers, resveratrol, curcumin, ER stress, autophagy, PI3K/AKT/mTOR

## Abstract

Curcumin and resveratrol are bioactive natural compounds displaying anti-inflammatory, anti-oxidant and anti-cancer properties. In this study, we compared the cytotoxic effects of these molecules and the molecular mechanisms involved against Her-2/neu-positive breast and salivary cancer cell lines. We found that both curcumin and resveratrol were efficient in reducing cancer cell survival and that they differently affected autophagy, ROS and activation of the PI3K/AKT/mTOR pathway. Moreover, we found that resveratrol and curcumin in combination exerted a stronger cytotoxic effect in correlation with the induction of a stronger ER stress and the upregulation of pro-death UPR molecule CHOP. This effect also correlated with the induction of pro-survival autophagy by curcumin and its inhibition by resveratrol. In conclusion, this study unveils new molecular mechanisms underlying the anti-cancer effects of resveratrol, curcumin and their combination, which can help to design new therapeutic strategies based on the use of these polyphenols.

## 1. Introduction

Human epidermal growth factor receptor Her-2 and the rodent counterpart neu (Her-2/neu) amplification/overexpression drives the malignant transformation and cell survival of several cancers, including breast, gastric pancreatic and salivary duct cancers, therefore, it represents an important target in anti-cancer therapy [1]. Interestingly, several dietary polyphenols, bioactive components of the Mediterranean diet, have been reported to downregulate Her-2/neu protein and suppress the oncogenic pathways activated downstream of it, such as phosphatidylinositol-4,5-bisphosphate 3-kinase/serine/threonine kinase/mechanistic target of rapamycin kinase (PI3K/AKT/mTOR) [2,3,4] and signal transducer and activation of transcription (STAT) 3 that contribute to their stem cell and epithelial to mesenchymal transition (EMT) marker expression [5]. Curcumin, a flavonoid originating from *Curcuma longa*, has been reported to have several beneficial properties, such as tumor prevention and cytotoxicity against cancers including those that are Her-2/neu positive [6,7,8,9,10]. The inhibition of multiple molecular pathways such as PI3K/AKT/mTOR, nuclear factor kappa B (NF-kB) and STAT3 have been reported to be involved in its anti-cancer effect even if all the pathways involved remain to be elucidated [11]. Among the mechanisms through which curcumin may reduce cancer cell survival there is reactive oxygen species (ROS) modulation, although both ROS reduction [12] and its increase [13,14] have been reported to contribute to their mediated anti-cancer effects. Indeed, it is known the ROS need to be well balanced in order to keep cells alive, as either their reduction or increase may perturb cellular homeostasis.

Resveratrol (*trans-*3,5,4′-trihydroxystilbene), a stilbene phytoalexin, found at high concentrations in grapes and red wine, similarly to curcumin, has been shown to exert multiple beneficial functions including the anti-cancer one [15]. However, in the case of resveratrol, the molecular mechanisms involved are even less clarified. The main problem with the clinical use of these natural compounds is their low bio-availability, and therefore, an active search is ongoing in the attempt to overcome this problem. The use of these natural molecules is strongly encouraged by their capacity to counteract the immune dysfunction induced by tumors [16,17] and restore immune response [16], which is needed for complete tumor eradication [18], and by their low toxicity. Resveratrol has also been shown to exert a cytotoxic effect against Her-2/neu cancers [19], and in combination with curcumin, its cytotoxicity can be even potentiated, either in vitro or in vivo in a Her-2/neu transgenic mouse model [9]. Both resveratrol and curcumin may interfere with autophagy, a degradative process that regulates cell differentiation [20,21] and helps to face to nutrient shortage and the stress derived from anti-cancer therapies [22,23]. Moreover, resveratrol [24] and curcumin [25] have been reported to perturb endoplasmic reticulum [26] homeostasis [27], activating the unfolded protein response (UPR), a process strictly interconnected with autophagy [28]. Indeed, UPR, orchestrated by the three main sensors inositol-requiring enzyme 1 alpha (Ire1alpha), PKR-like ER kinase (PERK) and activating transcription factor 6 (ATF6), leads to the upregulation of chaperone molecules such as glucose-regulated protein 78 (BIP/GRP78) and the promotion of protein degradation, helping cells to relieve stress. However, UPR can also promote apoptosis through the upregulation of C/EBP homologous protein (CHOP) when the adaptive capacity of UPR is overwhelmed.

Based on the above-reported background, in this study, we more deeply explored the molecular mechanisms leading to the anti-cancer effects of curcumin and resveratrol and their combination toward two murine cancer cell lines, TUBO (mammary) and SALTO (salivary gland) derived from Her-2/neu transgenic mice. We show that these compounds have different impacts on PI3K/AKT/mTOR activation, autophagy and ER stress/UPR and that the higher cytotoxic effect mediated by their combination correlated with the exacerbation of ER stress and the upregulation of the pro-death UPR molecule CHOP.

## 2. Results

### 2.1. Curcumin, Resveratrol and Their Combination Impair TUBO and SALTO Cell Survival

**Figure 1:**
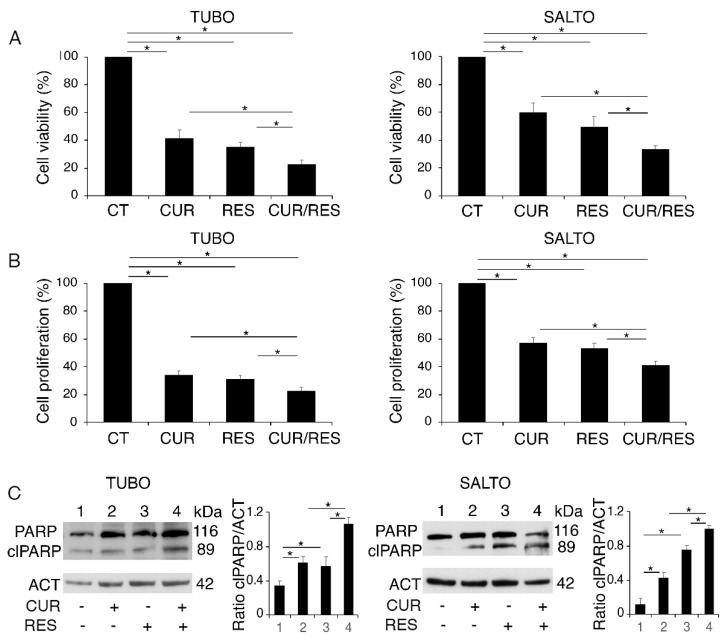
Curcumin (CUR), resveratrol [9] and their combination (CUR/RES) induce a cytotoxic effect in TUBO and SALTO cell lines. TUBO and SALTO cells were untreated (CT) or treated with curcumin (15 μM), resveratrol (15 μM) or combination of both for 48 h, and (**A**) cell survival and (**B**) cell proliferation were evaluated by trypan blue and MTT assays, respectively. Histograms represent the mean plus standard deviation (S.D.) of three independent experiments. (**C**) Western blot analysis showing cleavage PARP (clPARP) in TUBO and SALTO cells treated with curcumin, resveratrol or combination of both, as above reported. Histograms represent the mean plus S.D. of densitometric analysis of three independent experiments. Actin was used as loading control (ACT). One representative experiment is shown. * *p* value < 0.05.

We used two different cancer cell lines overexpressing Her-2/neu TUBO and SALTO, originated from breast and salivary glands cancers, respectively, to compare the cytotoxic effect of curcumin and resveratrol, either as single agents or in combination. We found that both compounds were able to impair cell survival in these cell lines after 48 h of treatment, and, according to previous studies [7,9], we confirmed that the cytotoxic effect was stronger when curcumin and resveratrol were used in combination. This was based on trypan blue (Figure 1A) and MTT (Figure 1B) assays, in which we also noticed that TUBO were more sensitive than SALTO cells to all treatments used. As shown in Figure 1C, these natural compounds, particularly when used in combination, induced apoptotic cell death in these cells, as indicated by the appearance of the cleaved form of poly(ADP-ribose) polymerase (PARP).

### 2.2. Curcumin and Resveratrol Differently Affect Autophagy in Both Cell Lines

**Figure 2:**
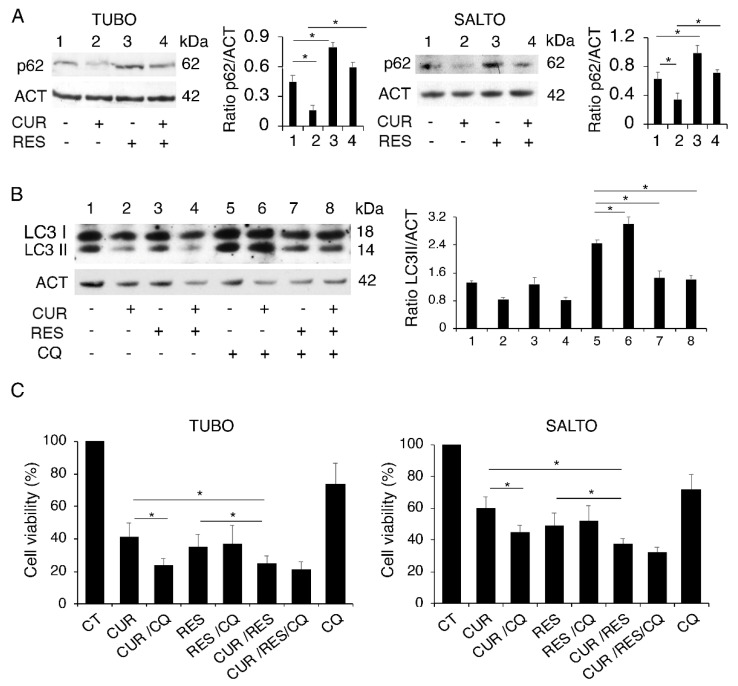
Curcumin promotes autophagy while resveratrol reduces this process also in combination with curcumin. (**A**) p62 expression level in TUBO in SALTO cells untreated or treated with curcumin, resveratrol or combination of both, at the above reported concentrations, for 48 h, as assessed by Western blot analysis. (**B**) LC3I/II expression was evaluated in TUBO cells treated by curcumin, resveratrol or combination of both in the absence or in the presence of chloroquine (10 µM) added for the last 10 h. One representative experiment is shown. Histograms represent the mean plus standard deviation of densitometric analysis of three independent experiments. Actin was used as a loading control. (**C**) Cell survival as evaluated by trypan blue assay in cells treated by curcumin, resveratrol or combination of both in the absence or in the presence of chloroquine. Histograms represent the mean plus standard deviation of three independent experiments. * *p* value < 0.05.

The activation of autophagy in the course of chemotherapy generally represents a mechanism that helps cells to adapt to stress. However, it has been reported that autophagy can also have a pro-death role, in some circumstances [29]. Therefore, it is important to investigate the impact of drugs on the autophagy to evaluate whether and how this process can be manipulated to improve the outcome of chemotherapeutic treatments. Here, we investigated this aspect and found that curcumin induced a complete autophagic flux while resveratrol reduced this process, also in combination with curcumin. This was indicated by the expression level of sequestosome (p62/SQSTM-1) (Figure 2A), a protein mainly degraded through this catabolic route, that was reduced by curcumin, while it accumulated in the presence of resveratrol. To better assess the regulation of autophagy by curcumin, resveratrol or their combination, the expression level of microtubule-associated protein 1A/1B-light chain 3 (LC3I/II) was evaluated by Western blot analysis, in the absence and in the presence of chloroquine (CQ). The latter, by preventing the degradation LC3II, allows us to evaluate LC3II formation, which is indicative of autophagy activation [30]. As shown in Figure 2B, the LC3II level accumulated in curcumin-treated cells in combination with chloroquine in comparison with those treated with chloroquine alone, confirming that this compound induced complete autophagy. Conversely, following resveratrol treatment, LC3II did not further increase in the presence of chloroquine, indicating that resveratrol reduced autophagy, as a single treatment and also in combination with curcumin (Figure 2B). We then evaluated the role of autophagy activation by curcumin and once again used chloroquine as an autophagy inhibitor. As shown in Figure 2C, the curcumin/chloroquine combination exerted a higher cytotoxicity compare to that of curcumin alone, while chloroquine did not further increase the cytotoxic effect of resveratrol and slightly increased that of the curcumin/resveratrol combination, according to the effects on autophagy mediated by these treatments.

These data suggest that resveratrol could act similarly to chloroquine in reducing the pro-survival autophagy induced by curcumin and, through this mechanism, potentiate the curcumin-mediated cytotoxic effect.

### 2.3. Curcumin and Resveratrol Have a Different Impact on PI3K/AKT/mTOR Activation

To evaluate the molecular mechanisms leading to the cytotoxic effect of curcumin and resveratrol and their different regulation of autophagy, we explored the activation of the PI3K/AKT/mTOR pathway, which is activated and involved in cell survival of Her-2/neu-overexpressing cancers [31].

**Figure 3:**
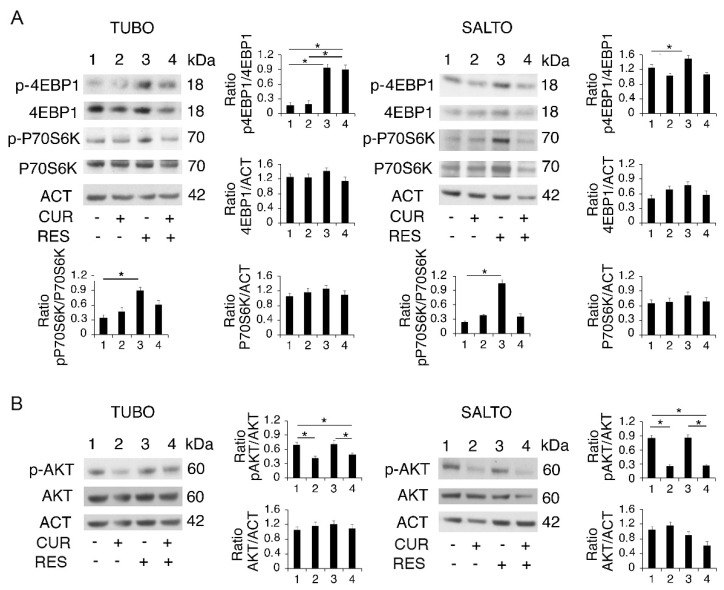
Curcumin and resveratrol affect PI3K/AKT/mTOR activation. (**A**) 4EBP1 and P70S6K mTOR targets and (**B**) AKT phosphorylation as evaluated by Western blot analysis in curcumin-, resveratrol- and curcumin/resveratrol-treated TUBO and SALTO cell lines, after 48 h of treatment. One representative experiment is shown. Histograms represent the mean plus standard deviation of densitometric analysis of three independent experiments. Actin was used as a loading control. * *p* value < 0.05.

With this aim, the activation of eukaryotic translation initiation factor 4E binding protein 1 (4EBP1) and P70 ribosomal protein S6 kinase B1 (P70S6K), mTOR complex 1 (mTORC1) targets, and of AKT, a kinase that acts upstream of mTORC1 and downstream of mTOR complex 2 (mTORC2), was evaluated [32]. As shown in Figure 3A,B, the phosphorylation of both mTORC1 targets increased following resveratrol treatment, and AKT phosphorylation was slightly affected by it. Curcumin and its combination with resveratrol instead slightly affected mTOR target phosphorylation, while they reduced that of AKT. The difference in regulation of the PI3K/AKT/mTOR pathway by curcumin and resveratrol may correlate with the regulation of autophagy mediated by these compounds, with the PI3K/AKT/mTOR pathway being central in the regulation of autophagy [33].

### 2.4. Curcumin and Resveratrol Dysregulate ROS in TUBO and SALTO Cell Lines

**Figure 4:**
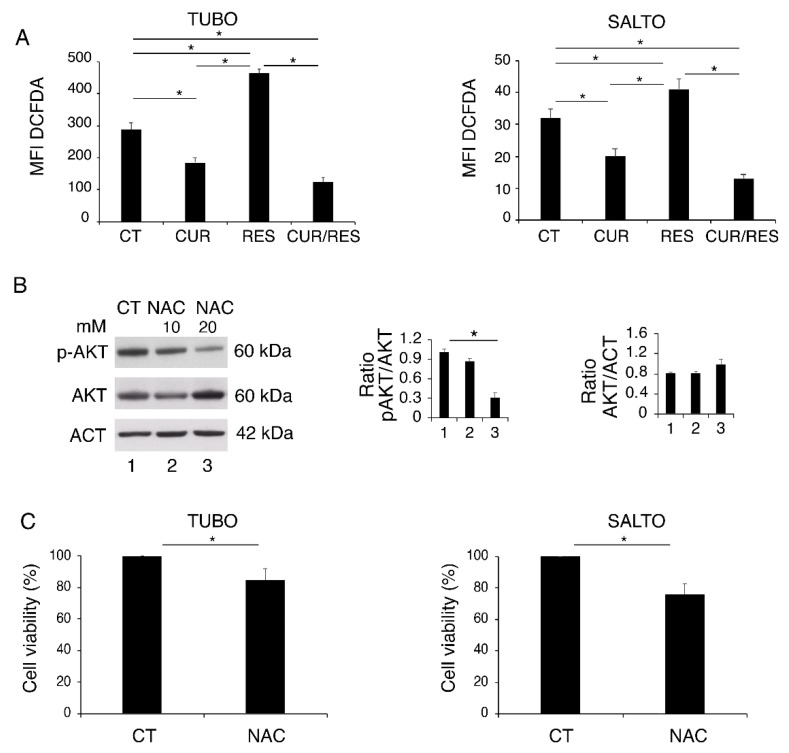
ROS modulation in curcumin-, resveratrol- and curcumin/resveratrol-treated cells. (**A**) Intracellular ROS levels after 48 h of curcumin, resveratrol and curcumin/resveratrol treatment, as evaluated by FACS analysis after DCFDA staining. Histograms represent the mean plus standard deviation of three independent experiments. (**B**) Dose-dependent effect of NAC (10 and 20 mM) on AKT phosphorylation in TUBO cells treated for 48 h with curcumin, resveratrol and curcumin/resveratrol. One representative experiment is shown. Actin was used as a loading control. Histograms represent the mean plus standard deviation of densitometric analysis of three independent experiments. (**C**) Cell survival as evaluated in TUBO cells treated by NAC (20 mM) for 48 h. Histograms represent the mean plus standard deviation of three independent experiments. * *p* value < 0.05.

Even if curcumin and resveratrol are known to be anti-oxidant compounds, they have been reported to either upregulate or downregulate ROS [27]. Interestingly, both effects may lead to a reduction of cancer cell survival, as a balanced level of ROS is required to sustain the activation of oncogenic pathways and maintain cell survival [34]. Here, we found that ROS levels increased following resveratrol treatment, while they were reduced by curcumin and by the combination of both (Figure 4A). To investigate whether ROS reduction could play a role in reducing the phosphorylation of AKT by curcumin and curcumin/resveratrol combination, we treated TUBO cells with the ROS-scavenger *N*-acetyl-l-cysteine (NAC) and found that the activation of AKT was reduced in a dose-dependent fashion (Figure 4B). Given the importance of AKT in sustaining cell survival, we also found that NAC was also able to reduce TUBO and SALTO cell survival (Figure 4C), suggesting that ROS reduction could contribute to curcumin- and curcumin/resveratrol-combination-mediated cytotoxicity. On the other hand, NAC treatment partially rescued cell survival of resveratrol-treated cells (data not shown), suggesting that ROS increase could play a role in resveratrol-mediated impairment of cell survival.

### 2.5. Curcumin/Resveratrol Combination Upregulates the PERK/eIF2alpha/CHOP Axis of UPR in Both TUBO and SALTO Cells

Polyphenols including resveratrol [24] and curcumin [25] may induce ER stress through different mechanisms, leading to UPR activation. Moreover, ER stress is strictly interconnected with autophagy, as it may contribute to the elimination of unwanted materials that cause ER stress [28]. Interestingly, ER stress is also strongly influenced by the activation status of AKT [35] and mTOR [36]. Depending on the intensity and duration of stress, UPR may shift the balance toward cell survival or death, by upregulating BIP or CHOP, respectively [37,38].

**Figure 5:**
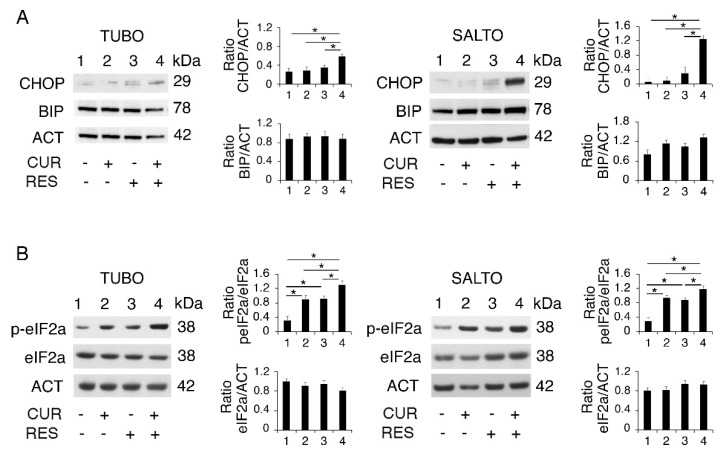
Curcumin and resveratrol combination activates the PERK/eIF2alpha/CHOP axis of UPR. (**A**) CHOP, BIP and (**B**) eIF2alpha activation was evaluated by Western blot in curcumin-, resveratrol- and curcumin/resveratrol-treated cells, as above reported for 48 h. One representative experiment is shown. Actin was used as a loading control. Histograms represent the mean plus standard deviation of densitometric analysis of three independent experiments. * *p* value < 0.05.

Therefore, we next investigated the impact on ER stress/UPR activation of curcumin, resveratrol and curcumin/resveratrol combination. We found that the expression level of the pro-death UPR molecule CHOP slightly increased following the resveratrol treatment, while it was strongly upregulated by the curcumin/resveratrol combination (Figure 5A). This effect may underlie the stronger cytotoxic effect induced by the curcumin/resveratrol combination and likely occurs in correlation with the inhibitory effect of resveratrol on curcumin-induced autophagy. Differently from CHOP, the pro-survival UPR molecule BIP was slightly affected by these treatments, as a slight reduction was observed only in curcumin/resveratrol-treated TUBO (Figure 5A) cells that were more susceptible to all the treatments used in this study.

As PERK is the UPR sensor most involved in CHOP upregulation [39], we then evaluated the activation of the PERK axis, by assessing the phosphorylation status of its target eukaryotic translation initiation factor 2 alpha (eIF2alpha). As shown in Figure 5B, eIF2alpha phosphorylation increased following treatment by curcumin and resveratrol and more strongly by the combination of both, mirroring the results observed for CHOP expression and suggesting a stronger ER stress induction by the curcumin and resveratrol combination. 

## 3. Discussion

Polyphenols represent a promising therapeutic strategy against inflammation-based diseases including cancer, although studies are still ongoing to better understand the pathways targeted by them and to improve their bioavailability and stability. As an effective approach to improve their availability, nanoencapsulation in o/w nanoemulsion-based delivery systems incorporating resveratrol and curcumin could be used. Piperine supplementation may also be a promising strategy to overcome the problem of the short half-life of these compounds [40]. The capacity of polyphenols to target multiple oncogenic pathways contributes to the anti-cancer effects of these compounds, e.g., the flavonoid curcumin [41] and the stilbene resveratrol [42]. This allows the prevention of the rebound effect that can occur when the inhibition of a single pathway may result in the hyperactivation of others, as a cell defensive mechanism. Last, but not least, the use of polyphenols is strongly encouraged by the fact that they not only are not toxic against cells of the immune system cells but rather also counteract the immune dysfunction that tumors usually induce [15]. Given the anti-cancer potential of these natural products, in this study, we investigated more in detail the molecular mechanisms leading to their cytotoxic effect, alone or in combination, against breast and salivary gland tumor cell lines derived from of Her-2/neu transgenic mice.

We found that curcumin, resveratrol and their combination dysregulated the PI3K/AKT/mTOR pathway, intracellular ROS, autophagy and ER stress/UPR both in TUBO and SALTO cell lines. In particular, ROS and AKT phosphorylation was reduced, and autophagy was promoted by curcumin, while resveratrol increased the phosphorylation of mTOR targets 4EBP1 and P70S6K. By using the ROS-scavenger NAC, we showed that ROS sustained AKT phosphorylation and cell survival, suggesting that ROS reduction was involved in the cytotoxic effect mediated by curcumin against TUBO and SALTO cells. Regarding resveratrol, its cytotoxicity instead correlated with the increase of ROS, which is known to be detrimental for cancer cell survival [43].

The inhibition of basal autophagy on which cancer cells rely to face their basal stress could contribute to the resveratrol-mediated cytotoxicity, as demonstrated by the use of chloroquine that per se partially reduced TUBO and SALTO cell survival. Moreover, resveratrol inhibited the pro-survival autophagy induced by curcumin, which resulted in an increased cytotoxic effect. Exploring the impact of drugs on autophagy and its role in cell survival is particularly important as autophagy inhibition may improve the outcome of anti-cancer treatments and also regulate the immunogenicity of cell death [29,44]. Interestingly, we have previously shown that chloroquine potentiated curcumin cytotoxicity in immune-deficient mice, while it counteracted its cytotoxic effect in immune-competent mice, in which an increase of T regulatory cell infiltration into the tumor bed was observed [7]. Conversely, the use of resveratrol, which we have shown here to be also able to reduce autophagy induced by curcumin similarly to chloroquine, was previously found to also potentiate curcumin cytotoxicity in vivo, in immune-competent mice [9]. This suggests that, at least in this case, resveratrol could be a better choice to be used in combination with curcumin compared to chloroquine, obtaining similar effects on autophagy and likely not dysregulating the immune response.

This study evidenced that the stronger reduction of cell survival induced by curcumin/resveratrol combination, besides the reduction of autophagy, contributed the increased expression of CHOP. This is indeed a pro-death UPR molecule upregulated in the course of a strong ER stress, such as when autophagy is inhibited during therapeutic treatments that are able to induce it [45,46]. CHOP may shift the UPR from an adaptive response toward cell death induction through several mechanisms such as the reduction of B-cell lymphoma-2 (Bcl-2) and the upregulation of Bcl-2 associated X (BAX) [47].

Understanding the interplay between autophagy and UPR and exploring the impact of drugs on such interplay is important as UPR may also be manipulated to promote cancer cell death [48]. This is even more important when considering that this strategy may allow the improvement of the outcome of anti-cancer therapies even against cancers carrying mutations in p53-encoding gene, which are known to be highly resistant to most cytotoxic treatments [49].

## 4. Materials and Methods

### 4.1. Cell Cultures and Treatments

Neu-overexpressing salivary gland cancer cells (H-2d) (SALTO-5), established from a salivary carcinoma arising in BALB-neuT transgenic male mice, were kindly provided by Prof. F. Cavallo (University of Turin, Turin, Italy) and Prof. P.L. Lollini (University of Bologna, Italy) [50]. BALB-neuT mammary cancer cells (H-2d) (TUBO) overexpressing activated rat ErbB2/neu were kindly provided by Prof. G. Forni (University of Turin, Turin, Italy) [51].

TUBO cells and SALTO cells were cultured in DMEM (Sigma-Aldrich, MO, USA), supplemented with 20% fetal bovine serum (FBS) (Sigma-Aldrich, MO, USA), L-glutamine (Aurogene, Rome, Italy) and streptomycin (100 μg/mL) and penicillin (100 U/mL) (Aurogene) in 5% CO_2_ at 37 °C. Cells were plated in six-well plates at a density of 3 × 10^5^ cells/well in 2 mL and treated singly or in combination with curcumin (CUR) (15 µM) (Sigma-Aldrich, MO, USA) (purity HPLC > 94%) and resveratrol (RES) (15 µM) (Sigma-Aldrich, MO, USA) (purity HPLC > 98%) diluted in DMSO or left untreated adding the same concentration of DMSO, for 48 h. To evaluate autophagic flux, in some experiments, cells were treated in the last 10 h of culture with chloroquine (CQ) (10 µM) (Sigma-Aldrich, MO, USA) (Figure 2). In some experiments, cells were treated for 48 h with ROS-scavenger *N*-acetyl-l-cysteine (NAC) or pre-treated for 1 h with NAC (10 and 20 mM) and then treated with resveratrol (RES) (15 µM) for an additional 48 h (Figure 4).

### 4.2. Trypan Blue Exclusion Assay

TUBO and SALTO cells were treated as previously described. After 48 h of treatment, a trypan blue (Sigma-Aldrich, MO, USA) exclusion assay was performed to test cell viability. Live cells were counted by light microscopy using a Neubauer Hemocytometer. The experiments were performed in triplicate and repeated at least three times.

### 4.3. MTT Cell Proliferation Assay

Cell proliferation was evaluated in TUBO and SALTO cells by *3*-[*4,5*-dimethylthiazol-2-yl]-2,5 diphenyl tetrazolium bromide (MTT) assay (Sigma-Aldrich, MO, USA). Briefly, 5 × 10^3^ cells/well were plated in 96-well plates in 100 µL of complete medium. The day after, cells were treated singly or in combination with curcumin (CUR) (15 µM) (Sigma-Aldrich, MO, USA) and resveratrol (RES) (15 µM) (Sigma-Aldrich, MO, USA) for 48 h. Untreated cells were used as control (CT). The MTT assay was performed following manufacturer’s instruction. The plates were analyzed with Absorbance 96 (Byonoy, Germany). The experiments were performed in triplicate and repeated three times.

### 4.4. Measurement of Intracellular Reactive Oxygen Species [50] Production

TUBO and SALTO cells were treated as previously described. After 48 h, to measure reactive oxygen species production, 10 μM of *2,7-*dichlorofluorescein diacetate (DCFDA; Sigma-Aldrich, MO, USA) was added to cell cultures for 15 min and, after washing in PBS, live cells, gated according to their forward scatter (FSC) and side scatter (SSC) properties, were analyzed by FACScalibur flow cytometer (BD Transduction Laboratories), using CellQuest Pro software (version 6.0, BD Biosciences, NJ, USA). For each analysis 10,000 events were recorded.

### 4.5. Western Blot Analysis

Cells, treated as reported above for 48 h, were washed in 1X PBS, lysed in RIPA buffer (150 mM NaCl, 1% NP-40, 50 mM Tris-HCl (pH 8), 0.5% deoxycholic acid, 0.1% SDS, protease and phosphatase inhibitors) and centrifuged at 14,000 rpm for 20 min at 4 °C. The protein concentration was measured by using the Bio-Rad Protein Assay (BIO-RAD laboratories GmbH, Munich, Germany), and 10 µg of protein was subjected to electrophoresis on 4–12% NuPAGE Bis-Tris gels (Life Technologies, UK) according to the manufacturer’s instruction. The gels were transferred to nitrocellulose membranes (Bio-Rad, Hercules, CA, USA) for 45 min in Tris-glycine buffer and the membranes were blocked in 1X PBS–0.1% Tween 20 solution containing 3% of BSA (Serva, Heidelberg, Germany), probed with specific antibodies and developed using ECL Blotting Substrate (Advansta, CA, USA).

### 4.6. Antibodies

To evaluate protein expression on Western blot membranes, the following antibodies were used: rabbit polyclonal anti-LC3 (1:1000) (Novus Biologicals), mouse monoclonal anti-p62 (1:500) (BD 610833), rabbit polyclonal anti-BiP (1:1000) (Cell Signaling), mouse monoclonal anti-CHOP (1:1000) (Cell Signaling), rabbit polyclonal anti-PARP (1:500) (Cell Signaling, 9542), rabbit monoclonal Phospho-Akt (Ser473) (1:500) (Cell Signaling #4060), rabbit monoclonal Akt (1:500) (Cell Signaling #9272), rabbit polyclonal anti Phospho-p70 S6 Kinase (Thr389) (1:500) (Cell Signaling #9234), rabbit polyclonal anti p70 S6 Kinase (1:500) (Cell Signaling #9202), rabbit monoclonal Phospho-4E-BP1 (Thr37/46) (1:500) (Cell Signaling #2855), mouse Monoclonal 4EBP1 (1:500) (Proteintech 60246-1-Ig), rabbit monoclonal Phospho-eIF2α (Ser51) (1:500) (Cell Signaling #3398), rabbit Monoclonal eIF2α (1:500) (Cell Signaling #5324), mouse monoclonal anti-β-actin (1:10000) (Sigma Aldrich) was used as loading control. The goat anti-mouse IgG-HRP (1:30000) (Bethyl Laboratories, A90-116P) and goat anti-rabbit IgG-HRP (1:30000) (Bethyl Laboratories, A120-101P) were used as secondary antibodies. All the primary and secondary antibodies were diluted in 1X PBS–0.1% Tween 20 solution containing 3% of BSA (Serva).

### 4.7. Densitometric Analysis

The quantification of proteins bands was performed by densitometric analysis using Image J software (1.47 version, NIH, Bethesda, MD, USA), which was downloaded from the NIH website (http://imagej.nih.gov, accessed on 10 January 2021).

### 4.8. Statistical Analysis

Results are represented by the mean ± standard deviation (S.D.) of at least three independent experiments, and a two-tailed Student’s t-test was used to demonstrate statistical significance. Difference was considered as statistically significant when *p*-value was at least <0.05.

## 5. Conclusions

This study unveils new molecular mechanisms underlying the anti-cancer effects of resveratrol, curcumin and their combination, which can help to design new therapeutic strategies based on the use of these polyphenols.

## Data Availability

Data is contained within the article.
